# Pharmacological inhibition of ABCC3 slows tumour progression in animal models of pancreatic cancer

**DOI:** 10.1186/s13046-019-1308-7

**Published:** 2019-08-05

**Authors:** Aleksandra Adamska, Alice Domenichini, Emily Capone, Verena Damiani, Begum Gokcen Akkaya, Kenneth J. Linton, Pierluigi Di Sebastiano, Xi Chen, Adam B. Keeton, Veronica Ramirez-Alcantara, Yulia Maxuitenko, Gary A. Piazza, Vincenzo De Laurenzi, Gianluca Sala, Marco Falasca

**Affiliations:** 1 Metabolic Signalling Group, School of Pharmacy and Biomedical Sciences, Curtin Health Innovation Research Institute, Curtin University, Perth, Western Australia 6102 Australia; 2 Dipartimento di Scienze Mediche, Orali e Biotecnologiche, University “G. d’Annunzio” di Chieti-Pescara, Centro Studi sull’Invecchiamento, CeSI-MeT, 66100 Chieti, Italy; 3 Queen Mary University of London, Barts and The London School of Medicine and Dentistry, Blizard Institute, Centre for Cell Biology and Cutaneous Research, Newark Street, London, E1 2AT UK; 4 Department of Surgery, Unit of Surgical Oncology, SS. Annunziata Hospital, G. D’Annunzio University, I-66100 Chieti, Italy; 5 Drug Discovery Research Center, USA Health Mitchell Cancer Institute, Mobile, AL USA

**Keywords:** Pancreatic ductal adenocarcinoma, ABC transporters, ABCC3, PDAC therapy, Tumour stroma

## Abstract

**Background:**

Pancreatic Ductal Adenocarcinoma (PDAC) is an aggressive and lethal disease, lacking effective therapeutic approaches. Available therapies only marginally prolong patient survival and are frequently coupled with severe adverse events. It is therefore pivotal to investigate novel and safe pharmacological approaches. We have recently identified the ABC transporter, ABCC3, whose expression is dependent on mutation of TP53, as a novel target in PDAC. ABCC3-mediated regulation of PDAC cell proliferation and tumour growth in vivo was demonstrated and was shown to be conferred by upregulation of STAT3 signalling and regulation of apoptosis.

**Methods:**

To verify the potential of ABCC3 as a pharmacological target, a small molecule inhibitor of ABCC3, referred to here as MCI-715, was designed. In vitro assays were performed to assess the effects of ABCC3 inhibition on anchorage-dependent and anchorage-independent PDAC cell growth. The impact of ABCC3 inhibition on specific signalling pathways was verified by Western blotting. The potential of targeting ABCC3 with MCI-715 to counteract PDAC progression was additionally tested in several animal models of PDAC, including xenograft mouse models and transgenic mouse model of PDAC.

**Results:**

Using both mouse models and human cell lines of PDAC, we show that the pharmacological inhibition of ABCC3 significantly decreased PDAC cell proliferation and clonal expansion in vitro and in vivo*,* remarkably slowing tumour growth in mice xenografts and patient-derived xenografts and increasing the survival rate in a transgenic mouse model. Furthermore, we show that stromal cells in pancreatic tumours, which actively participate in PDAC progression, are enriched for ABCC3, and that its inhibition may contribute to stroma reprogramming.

**Conclusions:**

Our results indicate that ABCC3 inhibition with MCI-715 demonstrated strong antitumor activity and is well tolerated, which leads us to conclude that ABCC3 inhibition is a novel and promising therapeutic strategy for a considerable cohort of patients with pancreatic cancer.

**Electronic supplementary material:**

The online version of this article (10.1186/s13046-019-1308-7) contains supplementary material, which is available to authorized users.

## Background

Pancreatic Ductal Adenocarcinoma (PDAC) is an aggressive malignancy with a dismal prognosis. In recent years, the incidence and mortality of PDAC is increasing, making PDAC the fourth most common cancer-related cause of death in the western world [1]. Five-year survival rates have not significantly improved in recent decades barely reaching 8%. The unfavourable prognosis is a multifactor consequence of high heterogeneity and aggressiveness of the tumours and lack of definitive symptoms usually leading to late-stage diagnosis. Moreover, there is a lack of effective therapeutic approaches. The main FDA-approved chemotherapeutics, gemcitabine, Abraxane and FOLFIRINOX give only a marginal improvement in patient survival and are frequently coupled with severe adverse effects [2]. High heterogeneity of PDAC tumours additionally restricts the design of effective targeted therapies [3]. Despite numerous clinical trials, so far only the Epidermal Growth Factor Receptor (EGFR) inhibitor, Erlotinib, has acquired FDA approval although its combination with gemcitabine provides a very marginal beneficial effect [4]. Novel pharmacological targets are therefore required whose activity regulates key signalling pathways in PDAC.

We have recently demonstrated that the ABC transporter ABCC3 (also known as MRP3) is an important regulator of PDAC signalling and that ABCC3 overexpression is a negative prognostic factor for PDAC patients [5]. We showed that ABCC3 expression is TP53-dependent, and we validated ABCC3 as a PDAC drug target by demonstrating that knockdown of ABCC3 markedly reduced PDAC cell proliferation and tumour growth in vivo [5]. By effluxing lysophosphatidylinositol (LPI), ABCC3 mediates the regulation of key signalling pathways suggesting that its inhibition should reduce PDAC progression. Thus, our data suggested the potential for pharmacological modulation of ABCC3 activity as a novel therapeutic approach in PDAC.

No studies have so far investigated the pharmacological targeting of ABCC3 beyond the reversal of chemoresistance. In this study, we explore the potential of ABCC3 inhibition to counteract PDAC progression. From a long-running medicinal chemistry/drug discovery process, we identified a novel small-molecule inhibitor of ABCC3, which reduces PDAC progression in vitro and tumour growth and disease progression in vivo in several animal models. Importantly, we show that ABCC3 inhibition not only affects PDAC tumours, but also targets PDAC stroma, which is enriched in ABCC3. Collectively, the results suggest that targeting ABCC3 with a small molecule inhibitor such as MCI-715 provides an effective therapeutic approach for a cohort of PDAC patients that overexpress ABCC3.

## Material and methods

### Cell culture

Cell lines were purchased from ATCC (VA, USA) and cultured as per manufacturer’s instructions in *Mycoplasma*-free conditions: AsPC1 (CRL-1682™), HPAFII (CRL-1997™), CFPAC-1 (CRL-1918™) and BJ (CRL-2522™). Human Immortalized Pancreatic CAF-stellate cells (CAFs) were purchased from Neuromics (Edina, MN, USA; #PC00B5). All cell lines were cultured less than 3 months after resuscitation and regularly tested for *Mycoplasma*. Primary cell line (KPC) was established from pancreatic tumours of KRAS^WT/G12D^, P53^WT/R172H^, PDX-1CRE^+/+^ (KPC) transgenic mouse model as described elsewhere [6–8].

### RNA interference

The role of ABCC3 in PDAC was assessed by transient knockdown with the use of siRNA transfection. For transient ABCC3 knockdown two siRNA sequences (siABCC3–1 (Hs_ABCC3_6), siABCC3–2 (Hs_ABCC3_15) QIAGEN) and control siRNA (siSCR) were used at working concentration of 75 nM. DarmaFECT 1 transfection reagent (Dharmacon) was used for the experiments according to manufacturer’s instructions. Western blotting was used to verify knockdown efficiency.

### Cell viability and colony formation assays

The effect of ABCC3 inhibition with MCI-715 on anchorage-dependent growth was assessed by trypan blue exclusion assay. Cells, seeded at a density of 5 × 10^4^ cells/well in 12-well or 2 × 10^4^ cells/well in 24-well cell culture plates, were treated in duplicate, with DMSO used as a negative control. After 72 h, viable cells were counted with trypan blue exclusion.

To validate the effect of MCI-715 on tumorigenic potential of cancer cells and their ability to form colonies in anchorage-independent conditions, soft agar colony formation assay was performed as previously described [8, 9]. Colonies were visualized with ChemiDoc XRS+ System (Bio-Rad) and quantified with ImageJ software. Each experiment was performed in three biologically independent replicates.

### Protein analysis

The evaluation of protein expression was performed by Western blot analysis. Cells and tissues were lysed in radioimmunoprecipitation assay (RIPA) buffer supplemented with Protease/Phosphatase Inhibitor Cocktail (Cell Signalling Technology) and sonicated. Proteins were separated by SDS-PAGE and detected by Western blotting according to standard procedures using the following antibodies (1:1000 in BSA 3%/TBST): ABCC3 (Santa Cruz, # sc-59612; Invitrogen, #PA5–23653), HIF1α (Novus Biologicals, #NB100–479), pSTAT3 Tyr705 (CST, #9131), GAPDH (CST, #5174), β-actin (CST, #4970), ⍺-actinin (CST, #3134), ⍺/β tubulin (CST, #2148), cleaved caspase 3 (CST,#9661), vimentin (CST, #5741), α-SMA (Abcam, #ab5694). Immunoblots were quantified using ImageJ and Image Lab 5.2.1 on the basis of three separate experiments.

### Two-colour Calcein transport assay and inhibition of ABCC3 by Sulindac and MCI-715

To verify the efficiency of ABCC3 blocking by sulindac and MCI-715, Calcein transport with and without ABCC3 inhibition was tested. Wild type ABCC3 cDNA encoded by recombinant pcDNA3.1 plasmid (pcDNA3-ABCC3) was a kind gift from Prof Susan Cole [10]. pDsRed2-C1 (pDsRed) was from Clontech (Mountain View, California, USA).

HEK293T cells were cultured as adherent monolayers in Dubecco’s Modifed Eagle Medium (DMEM) High Glucose (ThermoFisher scientific; Waltham, MA, USA) supplemented with 10% foetal calf serum (FCS). Transient transfection used polyethylenimine (PEI), as described previously [11]. Briefly, 6.25 × 10^5^ cells were seeded onto a T25 tissue culture flask and double transfected 24 h later with a transfection mix prepared from 7.5 μg pcDNA3-ABCC3 and 2.5 μg pDsRed in a 20 μl volume of 5% glucose and 17 μg of linear 25 kDa PEI (Sigma-Aldrich; Gillingham, Dorset, UK). The DNA/PEI complex was diluted in 5 ml DMEM and added to the cells. After a further 24 h the culture was supplemented with butyric acid to a final concentration of 2 mM to stimulate transcription. The cells were harvested after a further 24 h in versene and aliquots (2 × 10^5^ cells in 200 μl growth medium) and incubated with Calcein-AM (0.1 μM; Invitrogen, UK) with 0 μM to 750 μM inhibitor (Sulindac or MCI-715), for 20 min at 37 °C. Stock solutions of Sulindac and MCI-715 were prepared in DMSO; the vehicle had no effect on the transport assay (data not shown). The cells were then washed twice by pelleting at 160 x G and resuspended in 0.5 ml ice-cold DMEM minus phenyl red but supplemented with FCS to 1% v/v. The cells were analysed using a FACScan flow cytometer (Becton Dickinson, NJ, USA). The population was gated for 10,000 single cells of normal size and granularity. Calcein content was measured in the FL-1 (green) channel, and red fluorescence from the expressed DsRed was measured in the FL-2 channel. Flow cytometry data were acquired using CellQuest Pro Software (BD Biosciences, San Jose, CA) and analysed using FlowJo (Tree Star; OR, USA). ABCC3 transport activity was inferred from the fold difference in Calcein content of untransfected cells versus the transfected cells. To compare independent datasets the fold difference was normalised to 100% activity in the absence of inhibitor. Statistical analysis of the dose response from three biological replicate experiments was by GraphPad PRISM® V5.0 software with IC_50_ determined by non-linear regression analyses (Graphpad Software, CA, USA).

### Caspase 3/7 activity

Potential induction of apoptosis in PDAC cell lines after ABCC3 inhibition was verified by analysis of caspase 3/7 activity. PDAC cells were seeded in a 96-well cell culture plate at the density of 10.000 cells per well. The following day cells were treated with 10 μM MCI-715 and incubated with Incucyte Caspase 3/7 fluorogenic apoptosis detection reagent (1:1000) (Essen Bioscience) according to manufacturer’s instruction and monitored for up to 72 h using IncuCyte Life Cell Analysis Imaging System (Sartorius). The ratio between the increase in caspase 3/7 activity and cell confluence was calculated to assess the induction of apoptosis. Each experiment was performed in three biologically independent replicates.

### Cyclooxygenase assays

Cyclooxygenase activity was determined using the COX Fluorescent Inhibitor Screening Assay Kit (Cayman Chemical) according to the manufacturer’s recommendation. Recombinant COX-1 or COX-2 were incubated with MCI-715 for 20 min prior to the addition of arachidonic acid before initiating the assays. The fluorescent readout from the assay was measured using a Biotek Synergy H4 plate reader. Sulindac sulfide served as a positive control for the assay.

### Animal studies

All animal experiments were performed according to standard national and institutional guidelines. The Curtin University Animal Ethics Committee approved procedures on KPC mice (AEC_2016_40). Xenograft studies were approved by the Italian Ministry of Health (N.484/2016-PR). All animals were kept at 21 °C in ventilated cages cleaned weekly, with 12 h light/ 12 h dark cycle and provided with water and food ad libitum. Sample size was estimated on the basis of the power calculations performed previously in our group [9].

Female athymic CD-1 nu/nu mice (5–7 weeks old) from Charles River Laboratories (Calco, LC, Italy) were injected subcutaneously in the right flank with 3 × 10^6^ HPAFII cells in PBS (200 μl). When tumours reached a volume of 100 mm^3^ mice were randomized in groups of six animals and treated via oral gavage with 25 mg/kg of MCI-715 or vehicle (0.5% CMC/0.25% tween-80) three times a week, for three weeks. Mice were sacrificed when tumour volumes reached 1500 mm^3^. Patient-derived xenografts (PDXs) were established by engrafting samples of primary pancreatic cancer from patients after surgical resection into the right flank of 4–6-week-old female CD-1 nude mice. PDXs were passaged twice (P1 and P2) by sequential reimplantation in CD-1 mice. When tumours reached a volume of 50 mm^3^, animals bearing P2 PDXs were randomized into two groups (*n* = 6) and treated via oral gavage with 25 mg/kg of MCI-715 or vehicle three times a week, for three weeks and sacrificed as tumours reached 1000 mm^3^. Tumours were measured using a surgical calliper and volumes calculated according to the formula: tumour volume = (length * width^2^)/2.

KRAS^WT/G12D^, P53^WT/R172H^, PDX-1CRE^+/+^ (KPC) transgenic mice and control mice were maintained and genotyped by the Animal Research Centre (Murdoch, Western Australia) according to the original protocol [12]. All mice were maintained on a C57BL/6 genetic background. Both male and female mice were used for experiments. Mice were ear-marked and genotyped by the provider. After reaching 80 days (predicted time of the commencement of tumour development), KPC mice were palpated daily to assess tumour development. After the tumours reached palpable size animals were treated with 25 mg/kg MCI-715 (n = 6) or vehicle (*n* = 8) daily by oral gavage. Animals were monitored daily and sacrificed when visible signs of pain and distress could be observed, such as significant weight loss (more than 15% of initial bodyweight), dehydration, development of ascites and breathing problems caused by developing lymphoma or pain. Mice were sacrificed by snipping of the main cardiac vein followed by organ perfusion through the heart. Survival of mice was plotted using a Kaplan-Meier curve and quantified using a log rank test.

### Histology and immunohistochemistry

To verify whether the effects of ABCC3 inhibition on the signalling pathways, which activity was shown to be downregulated in vitro*,* are reflected in vivo, immunohistochemistry (IHC) analysis of murine tissues was performed.

Formalin fixed-Paraffin embedded (FFPE) pancreas and liver tissues were cut into 4 μm sections. A pathologist blinded to the experimental groups assessed histopathological analysis by Haematoxylin and Eosin (H&E) staining. IHC staining followed standard protocols after heat-induced antigen retrieval with pH 9.0 Tris/EDTA unless stated otherwise ABCC3 (Invitrogen, #PA5–23653; 1:25), pSTAT3 Y705 (CST, #9131; 1:400), HIF1α (Novus Biologicals, #NB100–479; 1:100), Vimentin (CST, #5741; 1:100, pH 6.0 Citrate buffer). Apoptosis was assessed using ApopTag® Plus Peroxidase in Situ Apoptosis Detection Kit based on the TUNEL method (Millipore). Web-based software ImmunoRatio was used for quantitative analysis. For each treatment arm, at least 15 photos were quantified for statistical analysis.

### Statistics

Sample size for each experiment was assessed based on previous work [9]. Statistical analysis was performed using GraphPad PRISM® V6.0 software (Graphpad Software, CA, USA); unpaired, two-tailed *t*-test (Western blot and IHC quantification), multiple t-test (tumour growth) and one-way ANOVA (cell growth) assuming independent samples and normal distributions were used. A 95% confidence interval was used for statistics and *P* < 0.05 was considered significant. Survival rates were described by a Kaplan-Meier curve and quantified by log rank (Mantel-Cox) test. Results are representative of at least three independent experiments and presented as mean ± SEM.

## Results

### A novel small molecule drug inhibits ABCC3 activity and reduces PDAC proliferation in vitro

We have recently demonstrated that ABCC3 plays a prominent role in stimulating PDAC cell and tumour growth, suggesting the potential of ABCC3 as a novel target in PDAC therapy [5]. We therefore sought to identify a specific inhibitor to validate ABCC3 as a pharmacological target for PDAC treatment. Sulindac is a nonsteroidal anti-inflammatory drug (NSAID) which inhibits cyclooxygenases involved in prostaglandin biosynthesis. Moreover, sulindac shows anticancer activities that may involve mechanisms unrelated to its cyclooxygenase inhibitory activity as previously reviewed [13]. Interestingly, it has also been reported to inhibit ABC transporters [14, 15]. A novel derivative of sulindac coded as MCI-715 was synthesized and chemically related to ADT-094 as previously reported [16]. MCI-715 was identified from a long-running synthetic chemistry/drug discovery program to reduce toxicity of sulindac by designing out COX inhibitory activity, while enhancing anticancer activity as previously described [17].

**Fig. 1:**
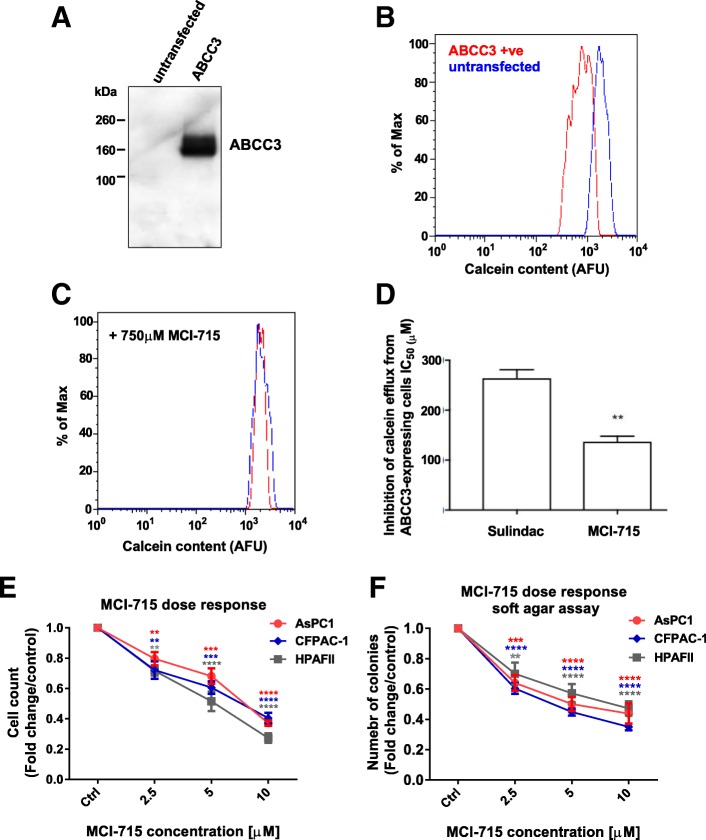
MCI-715 is a potent inhibitor of ABCC3 and its application significantly reduces PDAC cell growth. **a** Western blot probed with anti-ABCC3 antibody (C-18; Santa Cruz) confirming the overexpression of ABCC3 in transfected cells. Lane 1, whole cell lysate prepared from untransfected HEK293T; lane 2, whole cell lysate prepared from HEK293T cells transiently-transfected with pcDNA3.1-ABCC3. **b** ABCC3 effluxes Calcein-AM to reduce accumulation in HEK293T cells (left histogram). Flow cytometry histogram comparing Calcein accumulation in red-fluorescent dsRed-expressing HEK293T cells co-transfected with pcDNA3-ABCC3 (red trace) compared to non-expressing cells (blue trace). **c** ABCC3 is fully inhibited by 750 μM MCI-715 (right histogram; colour coding as before). **d** MCI-715 is a more potent inhibitor of calcein-AM efflux by ABCC3 than sulindac sulphide. IC_50_ data are presented as mean ± SEM of 3 independent experiments, statistical analysis was performed by unpaired Student’s t-test *p* = 0.0033; The effect of the treatment of indicated PDAC cell lines with increasing doses of MCI-715 on anchorage-dependent **e** and anchorage-independent **f** cell growth. PDAC cells were treated with indicated doses of MCI-715 and number of cells was assessed after 72 h. For the assessment of anchorage-independent growth, cells were plated on soft agar, treated with indicated concentrations of MCI-715 and number of colonies was counted after 4 weeks. Data is expressed as fold change of cells treated with vehicle (DMSO). All experiments are presented as mean ± SEM of 3 independent experiments. One-way ANOVA was used for statistical analysis, **p < 0.01, ***p < 0.001, ****p < 0.0001

The potency of MCI-715 to block ABCC3 activity was investigated. Calcein-AM is a hydrophobic dye that fluoresces in the green spectrum after the de-esterification of the aceto-methoxy moiety by cytoplasmic esterases. Calcein-AM is also a transport substrate of ABCC3 allowing the development of a live-cell assay to measure ABCC3 activity and inhibition by flow cytometry. ABCC3 was transiently expressed in otherwise naive HEK293T cells as shown by Western analysis (Fig. 1a). In the absence of an antibody to detect ABCC3 expression in live cells we co-transfected pDsRed (at a ratio of 1:3 (w/w) with pcDNA3-ABCC3) to generate a population of the competent cells that fluoresce in the red spectrum. The red-fluorescent (transfected) cells accumulated less Calcein than non-transfected cells indicative of efficient co-transfection with both plasmids (Fig. 1b). Incubation with a high concentration of MCI-715 completely abolished the difference in calcein accumulation between the ABCC3-expressing and non-expressing cells (Fig. 1c). By titration of the concentration of inhibitor, an IC_50_ for MCI-715 was determined and found to inhibit ABCC3 with 2-fold higher potency than the parent compound sulindac sulfide (Fig. 1d). This is not the only difference between sulindac and MCI-715. While sulindac sulphide, the active form of sulindac, inhibited COX-1 and COX-2 with IC_50_ values of 1.0 and 6.7 μM respectively (Additional file 1: Figure S1a), MCI-715 did not inhibit COX-1 or COX-2 above 50% even at a concentration of 100 μM (Additional file 1: Figure S1b). The pharmacokinetics of the two compounds are also different. In vivo plasma levels of MCI-715 following oral administration were approximately 10-fold higher, relative to sulindac, over a sustained period in mouse pharmacokinetic studies (Additional file 1: Figure S1c). These observations of increased potency against ABCC3, reduced COX antagonism and increased bioavailability suggest the potential for improved safety, efficacy and specificity of MCI-715 compared to sulindac sulphide as an ABCC3 inhibitor in PDAC therapy.

Validation of the potency of MCI-715 in counteracting PDAC cell growth was initially performed in vitro. Consistent with previous data from genetic downregulation of ABCC3 [5], a significant decrease in both anchorage dependent and independent growth was found after treatment of PDAC cells, characterized by enhanced expression of ABCC3, with increasing concentrations of MCI-715 (Fig. 1e,f; Additional file 1: Figure S1d). Interestingly, marginal effects of MCI-715 were noted in the PDAC cells with low expression of ABCC3 (SW1990, Additional file 1: Figure S1d, e), suggesting the specificity of MCI-715 towards ABCC3.

These data demonstrate the pharmacological potential of ABCC3 inhibition in decreasing PDAC cell proliferation and reducing their tumorigenic potential and clonal expansion, supporting ABCC3 as a novel target in counteracting PDAC progression. Together with in vitro experiments showing improved potency of MCI-715 to inhibit tumour cell growth relative to sulindac sulphide, these studies supported in vivo evaluation of the antitumor efficacy of MCI-715.

### Pharmacological inhibition of ABCC3 with MCI-715 inhibits STAT3 signalling and induces PDAC cell apoptosis

**Fig. 2:**
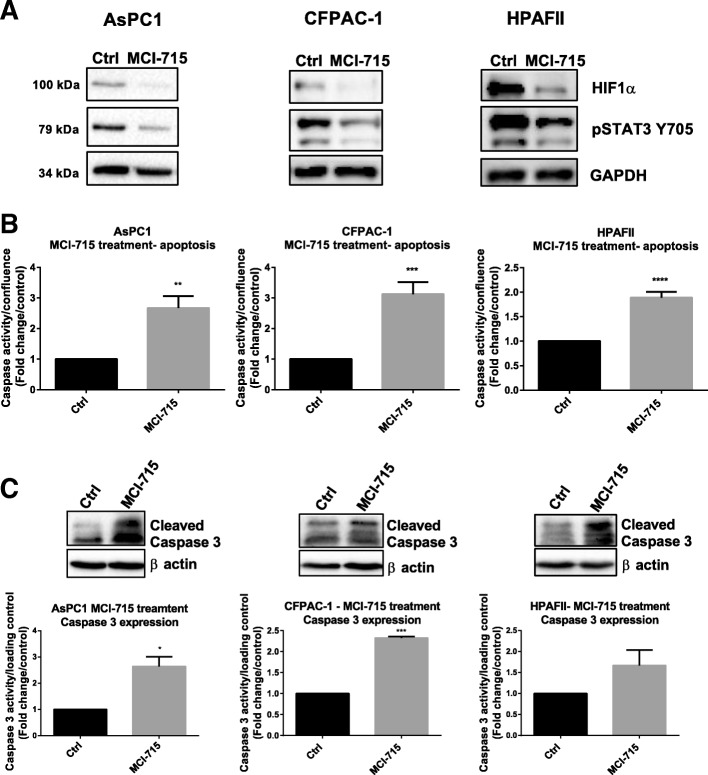
Pharmacological inhibition of ABCC3 reduces cell proliferation through STAT3 and HIF1α dysregulation and induction of apoptosis. **a** Representative Western blot images show the effects of pharmacological inhibition of ABCC3 with MCI-715 on the expression of pSTAT3 Y705 and HIF1α in three PDAC cell lines (AsPC1, HPAFII, CFPAC-1). Cells were treated with MCI-715 at the concentration of 10 μM and collected after 24 h (CFPAC-1) or 48 h (AsPC1, HPAFII). The quantitative analysis of *n* = 3 separate experiments is presented in Additional file 1: Figure S2; **b** The effects of the treatment of AsPC1, HPAFII and CFPAC-1 cell lines with 10 μM MCI-715 on the Caspase 3/7 activity (72 h post treatment) measured with Caspase 3/7 fluorigenic probe; **c** Representative Western blotting images and quantitative analysis of cleaved caspase 3 expression following treatment of indicated PDAC cell lines with 10 μM MCI-715. All results are presented as mean ± SEM of 3 independent experiments. The quantitative analysis was performed with the use of ImageJ and Image Lab software, unpaired Student’s t-test was performed for statistical analysis, *p < 0.05, **p < 0.01, ***p < 0.001, ****p < 0.0001

Considering the remarkable results obtained with ABCC3-targeting approach, the mechanisms of action of MCI-715 were more extensively explored. We have recently shown that ABCC3 exerts its functions through regulation of STAT3 and HIF1α pathways, as shown through knockdown of ABCC3. Due to the essential role played by these pathways in PDAC development and progression, several attempts have been made to inhibit their activity and thereby to slow down cancer progression [18, 19]. However, most of the preclinical or clinical validation failed due to the lack of efficacy and severe side effects of tested inhibitors [2]. Given the efficacy of MCI-715 in reducing PDAC cell growth and clonal expansion, we determined the potential effects of MCI-715 on STAT3 and HIF1α signalling. MCI-715 significantly reduced pSTAT3 Y705 and HIF1α levels in human PDAC cell lines (AsPC1, HPAFII, CFPAC-1) (Fig. 2a, Additional file 1: Figure S2), consistent with the effects of ABCC3 genetic knockdown [5]. The simplest interpretation of these data is that ABCC3 inhibition with MCI-715 reduces the activity of these oncogenic signalling pathways. It is well known that cancer cells develop mechanisms that enable them to escape programmed death (apoptosis) and survive in unfavourable conditions. Thus, a majority of therapeutics aim to induce apoptosis in cancer cells. Analysis of the activity of Caspase 3/7 in live cells by fluorogenic probe (Fig. 2b) and Western blot analysis of cleaved caspase 3 (Fig. 2c) demonstrated increased apoptosis following cell treatment with MCI-715. These results parallel the outcomes recently demonstrated after ABCC3 knockdown in the same cell lines [5], strongly suggesting that the observed effects of MCI-715 treatment are due to ABCC3 inhibition.

Collectively, our data suggest that the mechanisms of action of MCI-715 involve inhibition of STAT3 and HIF1α signalling via blockade of ABCC3, coupled with induction of apoptosis in the cells and reduced proliferation of PDAC cells.

### Pharmacological inhibition of ABCC3 with MCI-715 significantly reduces PDAC progression in mouse models

We have recently reported that downregulation of ABCC3 with the use of a specific shRNA decreased PDAC cell growth in vitro and importantly, significantly reduced tumour growth in a xenograft mouse model [5]. Following in vitro validation of ABCC3 pharmacological inhibition, MCI-715 antitumor activity was tested in vivo, using three different mouse models, to verify if the results obtained with ABCC3 knockdown could be reproduced using a pharmacological approach.

**Fig. 3:**
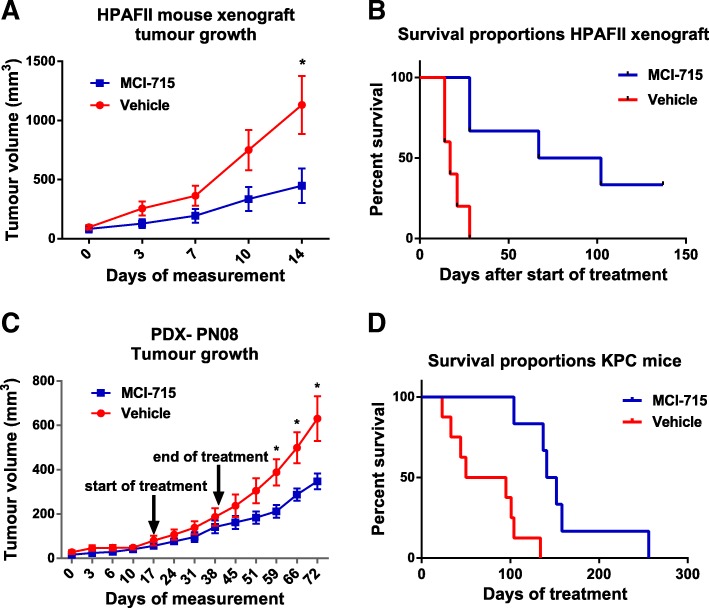
ABCC3 pharmacological inhibition reduces PDAC progression in in vivo mouse models. Mice were injected with HPAFII cells and treated with 25 mg/kg MCI-715 for 28 days as described in Methods section. Data presents the comparison of the tumour growth (**a**) and survival (**b**) of HPAFII xenograft mouse model treated with vehicle (*n* = 4) and MCI-715 (*n* = 6), Multiple-t-test was performed for statistical analysis of tumour growth, *p* = 0.0343, Logrank (Mantel-Cox) was performed for statistical analysis of the survival, p = 0.0033; (**c**) PDX mouse model was created and treated with 25 mg/kg MCI-715 as described in Methods section. Tumour growth in PDX mouse model of pancreatic cancer treated with vehicle (*n* = 5) and MCI-715 (n = 5) shows significant difference between the two treatment groups. Arrows indicate start and the end of the treatment period, Multiple-t-test was performed for statistical analysis of tumour growth *p < 0.05; (**d**) KPC transgenic mouse model was treated with 25 mg/kg MCI-715 as described in Methods section. Kaplan Meier survival curve of KPC mice treated with vehicle (*n* = 8) and MIC-715 (n = 6). Logrank (Mantel-Cox) test was performed for statistical analysis, *p* = 0.0010

A dose of 25 mg/kg MCI-715 was established as an efficacious and safe dose for the animal studies. Mice bearing HPAFII xenografts, as well as patient-derived xenografts (PDX) were treated with 25 mg/kg MCI-715 by oral gavage and the tumour volume was measured every three days. A statistically significant reduction in tumour growth was observed in MCI-715 treated HPAFII xenografts compared to the vehicle treated group (*p* = 0.0343) (Fig. 3a). Notably, this trend continued beyond the end of the treatment and complete tumour remission was noted in two out of six of the MCI-715 treated mice, a phenomenon not usually observed in PDAC xenograft models (Additional file 1: Figure S3a). Accordingly, significant extension of survival of treated mice was achieved (median survival: 17 days for vehicle vs 84.5 days for MCI-715; *p* = 0.0033) (Fig. 3b), with no evident side effects (Additional file 1: Figure S3b). Similarly, a significant reduction in tumour growth (*p* < 0.05) that resulted in an increased survival (not shown) was observed in PDX mice treated with MCI-715 (Fig. 3c), confirming the efficacy of MCI-715.

A transgenic mouse model of pancreatic cancer KPC (Kras^LSL.G12D/+^; p53^R172H/+^; PdxCre^tg/+^), closely mimicking human disease [12], was also used to evaluate the antitumor activity of MCI-715. The KPC model is characterized by close reproduction of the PanIN lineage, with 100% penetrance, dense stroma formation and fully developed tumours with pathology resembling human PDAC. Thus, the KPC model is a valuable tool in the preclinical validation of the anti-tumorigenic agents in PDAC therapy. Mice were palpated daily for the assessment of tumour development and once palpable tumour size was confirmed, mice were randomized into vehicle (*n* = 8) and MCI-715 (*n* = 6) treatment groups. Mice were treated daily with 25 mg/kg MCI-715 by oral gavage. Strikingly, a statistically significant increase in survival was achieved with MCI-715 treatment compared to vehicle-treated mice (*p* = 0.0010), (Fig. 3d). A two-fold increase in lifespan was observed from 72.5 days (median survival) in the control group to 146.5 days in the treatment group and, importantly, no sign of toxicity was apparent for MCI-715 treatment as assessed by body weight gain during the experiment and necropsy observations (Additional file 1: Figure S3c).

The data indicate the potential for strong efficacy and tolerability of MCI-715 to suppress PDAC progression in three different mouse models of pancreatic cancer, which provides the basis for further evaluation of MCI-715 and its derivatives as therapeutic agents.

**Fig. 4:**
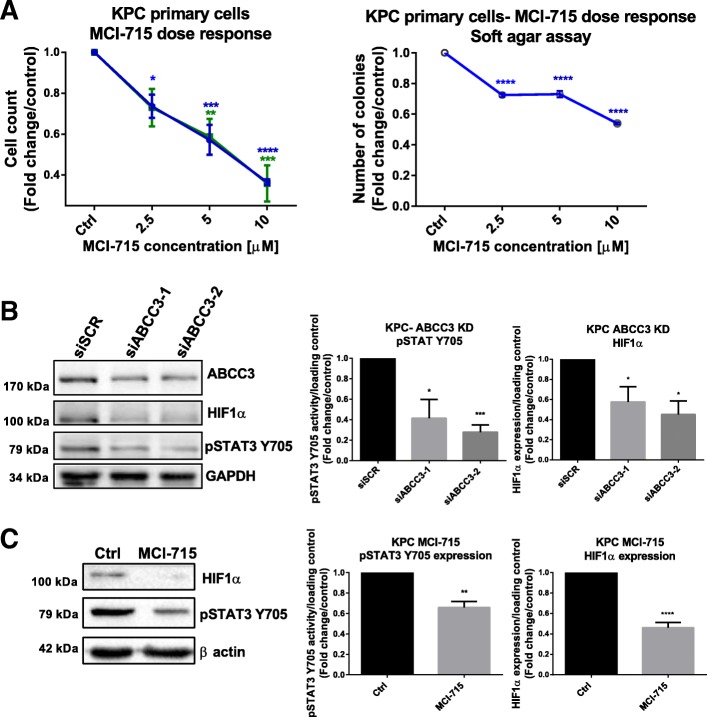
Targeting ABCC3 in KPC primary cell line reduces cell proliferation through STAT3 and HIF1α dysregulation. Primary cell lines were established from the pancreatic tumours of KPC mice as described in the Methods section. KPC cells were treated with indicated doses of MCI-715 and number of cells was assessed after 72 h. For the assessment of anchorage-independent growth, cells were plated on soft agar, treated with indicated concentrations of MCI-715 and number of colonies was counted after 4 weeks. **a** Data presents the effects of MCI-715 dose response treatment in established KPC primary cell lines on anchorage dependent and independent cell growth. Blue: I^st^ established cell line, Green: 2nd established cell line. Data is expressed as fold change of cells treated with vehicle (DMSO). The results are presented as mean ± SEM of 5 (blue) and 3 (green) independent experiments, One-was ANOVA was performed for statistical analysis, *p < 0.5, **p < 0.01, ***p < 0.001, ****p < 0.0001; Representative Western blot images showing the effects of knockdown of ABCC3 with two specific siRNAs **b** and treatment with 10 μM MCI-715 **c** on the expression of pSTAT3 Y705 and HIF1α in the KPC primary cell line. Cells were collected 48 h post transfection/treatment. Quantitative analysis is presented as mean ± SEM of 3 (pSTAT3 Y705) and 4 (HIF1α) independent experiments and expressed as fold change of expression in control sample in reference to loading control; unpaired Student’s t-test was performed for statistical analysis, *p < 0.05, **p < 0.01, ***p < 0.001, ****p < 0.0001

**Fig. 5:**
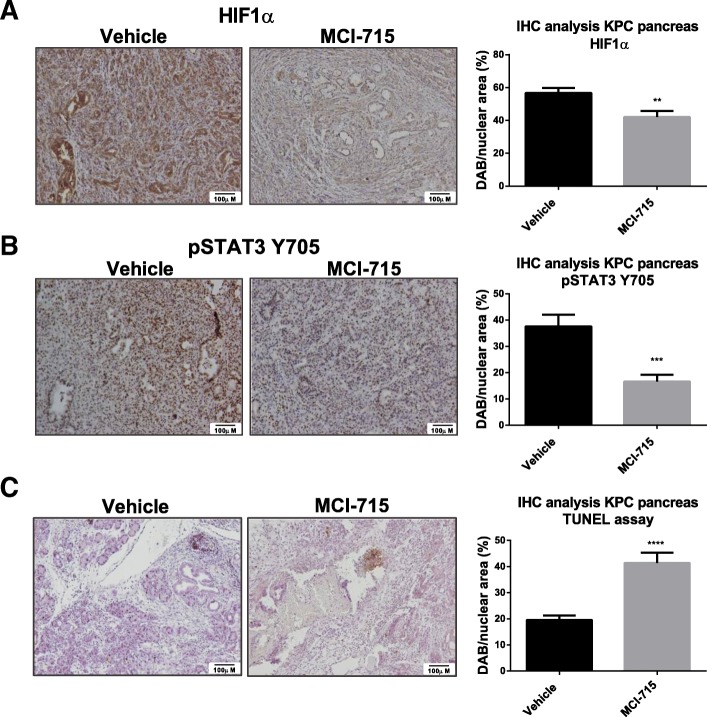
Pharmacological inhibition of ABCC3 dysregulates STAT3 and HIF1α signaling and induces apoptosis in vivo. Tumour tissues were resected for the KPC mice enrolled to MCI-715- treated or vehicle-treated group at the end of experiment. Protein expression in vivo was verified by IHC staining as described in Methods section. Data shows representative IHC staining of FFPE pancreatic tumor tissues resected from KPC mice showing differential expression of pSTAT3 Y705 (**a**) and HIF1α (**b**) between vehicle and MCI-715- treated mice samples. Scale bar: 100 μm. The quantitative analysis of IHC staining was performed with the use of ImmunoRatio software and the results are presented as mean ± SEM of 17 (Vehicle group) and 14 (MCI-715 group) different images from at least 4 different animals; unpaired Student’s t-test was performed for statistical analysis, **p < 0.01, ***p < 0.001; (**c**) Representative image of the TUNEL assay performed on FFPE pancreatic tissues resected from KPC mice treated with vehicle and MCI-715 (Scale bar: 100 μm). The quantitative analysis of IHC staining was performed with the use of ImmunoRatio software as mean ± SEM of 17 (Vehicle group) and 21 (MCI-715 group) images; unpaired Student’s t-test was performed for statistical analysis, **** p < 0.0001

To corroborate the data ex vivo and investigate the mechanism of action, a KPC primary cell line was established from murine tumours. MCI-715 treatment of primary KPC cells inhibited anchorage-dependent and -independent cell growth (Fig. 4a), confirming the efficacy of MCI-715 to block proliferation in the KPC mouse model. Moreover, decreased levels of both pSTAT3 Y705 and HIF1α were detected following both siRNA knockdown of ABCC3 (Fig. 4b) and its inhibition with MCI-715 in KPC primary cells (Fig. 4c), reconfirming the involvement of ABCC3 in regulation of activity of both pathways in this model. Additionally, IHC analysis of KPC mice tissues revealed a decrease in pSTAT3 Y705 and HIF1α levels following MCI-715 therapy, although the difference in HIF1α localization between MCI-715 and vehicle treated mice was more apparent by a decrease in stromal expression of the protein (Fig. 5a, b). Our findings thus suggest that pharmacological inhibition of ABCC3 with MCI-715 represents a novel mechanism for reducing the activity of STAT3 and HIF1α pathways that represent key oncogenic drivers of PDAC progression.

The TUNEL assay performed on paraffin-embedded mice tumour tissues revealed elevated levels of apoptotic cells in the tumours resected from MCI-715 treated mice compared to vehicle treated controls (Fig. 5c). Induction of apoptosis was also demonstrated in the KPC cell line upon both knockdown of ABCC3 and its pharmacological inhibition with MCI-715 as shown by Western blot analysis of cleaved caspase 3 (Additional file 1: Figure S4a). Similarly, reduced expression of Bcl-xl, a protein of anti-apoptotic function, located downstream of STAT3 signalling, was detected both by Western blot analysis of the same samples (Additional file 1: Figure S4b) and by immunohistochemistry (IHC) staining of the pancreatic tissues resected from KPC mice treated with MCI-715 or vehicle (Additional file 1: Figure S4c).

These data indicate that modulation of ABCC3 activity strongly influences PDAC progression both in vivo and ex vivo through mechanisms involving induction of apoptosis and downregulation of STAT3 and HIF1α signalling in the KPC model of PDAC.

### ABCC3 inhibition mediates stroma reprogramming

**Fig. 6:**
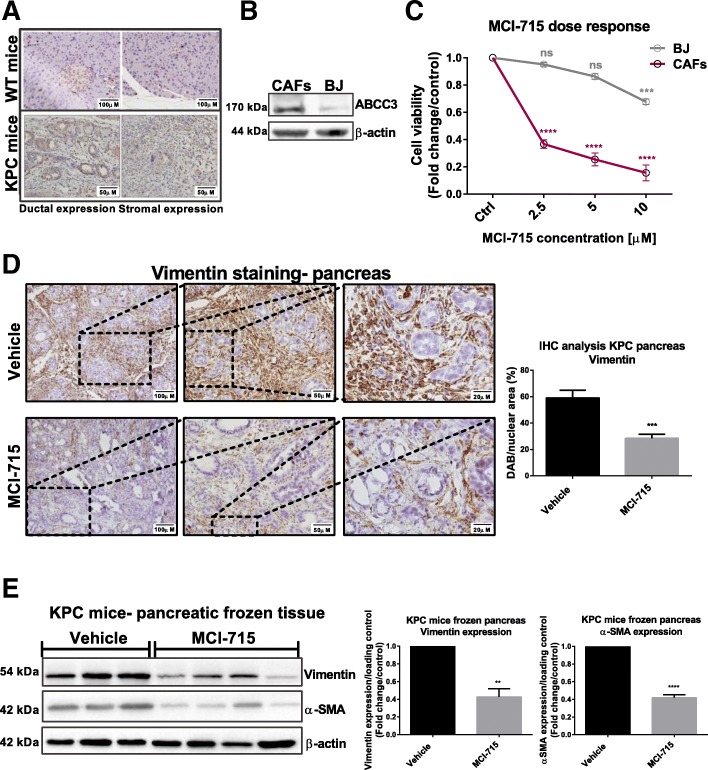
ABCC3 as an important stroma component. **a** Representative IHC staining of ABCC3 expression in the pancreas of KPC control (scale bar: 100 μm) and KPC (scale bar: 50 μm) mice showing ductal (left) and stromal (right) expression of ABCC3 in KPC mice; **b** Western blot analysis comparing ABCC3 expression in CAFs (cancer-associated fibroblasts) and BJ (normal fibroblasts) cells; **c** CAFs and BJ cells were treated with indicated doses of MCI-175 and number of cells was counted after 72 h. Data presents the effect of MCI-715 treatment on the viability of CAFs and BJ cells presented as mean ± SEM of 3 independent experiments. Data is expressed as fold change of cells treated with vehicle (DMSO), one-was ANOVA was used for statistical analysis, ***p < 0.001, ****p < 0.0001; **d** Representative IHC staining comparing vimentin expression in FFPE pancreatic tissues from KPC mice treated with vehicle or 25 mg/kg of MCI-715 (Scale bar: 100 μm, 50 μm, 20 μm). Quantitative analysis is presented as mean ± SEM of 8 (Vehicle) and 12 (MCI-715) separate images ***p < 0.001; **e** Western blotting analysis of vimentin and αSMA expression in snap frozen pancreatic tissues from KPC mice treated with vehicle (*n* = 3) or 25 mg/kg MCI-715 (*n* = 4), quantitative analysis was performed with the use of ImageJ software, unpaired Student’s t-test was performed for statistical analysis, **p < 0.01, ****p < 0.0001

Analysis of tumour tissues dissected from the KPC mice also demonstrated overexpression of ABCC3. High ABCC3 expression was observed not only in the tumour ducts, but also in the stroma of KPC mice tissue samples, suggesting a potential role of ABCC3 in the tumour microenvironment (Fig. 6a). The dense stroma surrounding PDAC tumours is a very complex environment composed of fibroblasts, stellate cells, immune cells and extracellular matrix (ECM) proteins and constitutes almost 80% of the tumour mass. PDAC stroma forms a barrier around the epithelial cells, restricting the delivery of applied therapeutics. In addition, tumour-stroma crosstalk has been recently demonstrated in PDAC [20, 21]. The interactions of the epithelial cells with surrounding stroma form a feed-forward loop, which perpetuates tumour growth in vitro and in xenograft mouse models [22, 23]. Thus, targeting stromal factors and reprogramming PDAC stroma activity is a potential therapeutic approach in PDAC treatment.

To verify the expression and the potential role of ABCC3 in human PDAC stroma, human immortalized cancer-associated fibroblasts (CAFs) were analysed. Western blot analysis of CAFs demonstrated increased ABCC3 expression in stromal cells compared to normal fibroblasts (BJ) (Fig. 6b). Additionally, MCI-715 efficacy in CAFs was significantly enhanced compared to normal fibroblasts, which were mostly unresponsive (Fig. 6c). These data corroborate the activity of MCI-715 towards ABCC3-expressing cells and demonstrate that targeting ABCC3 not only decreases cancer cell proliferation, but also likely affects ABCC3-expressing stromal cells.

Analysis of established KPC primary cells provided more insight into the importance of ABCC3 in cancer-stroma interactions. Phenotypically, the primary cells adopted a tumour-like morphology with the formation of epithelial clusters surrounded by a matrix of fibroblast-like cells, demonstrating the presence of both cell types in the established cell line. Thus, the effects of ABCC3 downregulation on both epithelial tumour cells and associated fibroblast may be assessed in this cell line. ABCC3 knockdown with siRNA significantly lowered cancer cell number and more strikingly, reduced the surrounding fibroblast-like cells (Additional file 1: Figure S5a). Similarly, treatment of the cells with MCI-715 reduced the viability of both cell types (Additional file 1: Figure S5b). These data reinforce the hypothesis of the expression and active role of ABCC3 in both PDAC ducts and stroma. Moreover, IHC analysis of tumour tissues from the KPC mice, showed a clear reduction in surrounding stroma, demonstrated by decreased vimentin levels in tissues from MCI-715-treated mice (Fig. 6d). Reduction of vimentin expression was additionally confirmed by Western blot analysis of frozen pancreatic tissues from KPC mice treated with MCI-715 (*n* = 4) versus vehicle (*n* = 3) (Fig. 6e). Similarly, genetic knockdown of ABCC3 and treatment of the primary cell line with MCI-715 reduced the expression levels of α-SMA and vimentin, in agreement with the hypothesis that ABCC3 is involved in both PDAC tumour and stroma development (Additional file 1: Figure S5c). These results suggest that ABCC3-targeted therapies might have dual effect in decreasing tumour bulk and targeting tumour-surrounding stroma.

## Discussion

In our latest paper we have discussed the involvement of ABCC3 in the regulation of PDAC progression [5]. ABCC3 activity-dependent control of anchorage-dependent and independent growth of PDAC cells was shown to be mediated by the regulation of STAT3 activity and apoptosis. More importantly, tumour growth in a xenograft mouse model was remarkably affected by the presence of active ABCC3. These data suggested ABCC3 as a novel pharmacological target in PDAC.

Here we have explored the potential of ABCC3 as a novel druggable target in PDAC. Due to the lack of effective and specific inhibitors of ABCC3, we investigated possible molecules with ABCC3-inhibitory activity. Sulindac is a nonsteroidal anti-inflammatory drug (NSAID) showing anticancer activities that may involve mechanisms unrelated to its cyclooxygenase inhibitory activity, including the blocking of several ABC transporters [13]. However, evidence suggests that the potential anticancer activity of sulindac cannot be attributed to the targeting and inhibition of cyclooxygenase alone. After careful consideration, we have conducted a medicinal chemistry library screening in order to identify sulindac derivatives that lack cyclooxygenase inhibitory activity, and with an enhanced anticancer activity. The result of this screening was the synthesis of a sulindac derivative, referred to as MCI-715, with a potent anticancer activity that has been investigated in this paper. We successfully showed that MCI-715 is a potent inhibitor of ABCC3, exhibiting two-fold higher efficiency than sulindac, but lacks COX inhibition. MCI-715 action mirrors the effects of reducing expression of ABCC3 by siRNA knockdown and significantly slows PDAC progression in vivo, whilst showing no adverse side effects. Using different mouse models of pancreatic cancer, we showed that MCI-715 treatment significantly reduced xenograft tumour growth and increased mouse survival rates. MCI-715 treatment also resulted in a striking two-fold increase in survival in the transgenic KPC mouse model of PDAC. The KPC mouse is the most physiologically relevant mouse model of pancreatic cancer, which reproduces the human pathology and progression of pancreatic cancer, therefore providing important pre-clinical validation that it is feasible to inhibit ABCC3 with a small molecule such as MCI-715. We have recently demonstrated that ABCC3 is able to transport lysophosphatidylinositol (LPI) in PDAC samples and that blocking of ABCC3 significantly reduced the levels of detected LPI [5]. LPI is a bioactive lipid, which mitogenic functions have been confirmed in several cancer types [24–27]. Our recent data showed that in PDAC, LPI activates one of G protein-coupled receptors, GPR55, triggering the activation of signalling cascades, leading to increased PDAC cell proliferation, clonal expansion and, consequently, disease progression. We showed that blocking of GPR55 significantly reduced LPI-induced tumorigenic functions and prolonged survival in the transgenic KPC mouse model [9]. Here we showed that pharmacological inhibition of ABCC3 strikingly reduces the progression of PDAC in several animal models of PDAC. We might speculate that the reduction of the levels of LPI in the tumour environment and consequently, reduced activation of GPR55 might contribute to observed effects. Moreover, ABCC3-mediated LPI release was shown to regulate STAT3 signalling, which constitutive activation has been reported to negatively correlate with the survival of PDAC patients. These data presented a novel mechanism of regulation of STAT3 in pancreatic cancer. Here we demonstrated that blocking of ABCC3 with MCI-715 inhibits STAT3 and HIF1α signalling pathways, key regulators of PDAC development and progression. Based on our findings, we speculate that the remarkable effects observed in animal models of PDAC after ABCC3 pharmacological modulation are due in part to the indirect inhibition of STAT3 pathway and hypoxia both in the tumour and surrounding stroma. Given the failure of the majority of clinical trials aiming at direct targeting of STAT3 or PDAC stroma [2], our results provide the possibility for a new effective and safe therapeutic approach.

Although our data indicate the activity of MCI-715 towards ABCC3, other ABCC transporters, especially ABCC1, share a similar structure and may exhibit redundant functions to ABCC3, suggesting a possible cross-reactivity of MCI-715 with other ABCC transporters. In fact, as sulindac is known to have activity against ABCC1, it is reasonable to hypothesise that sulindac derivatives, as MCI-715 may also target ABCC1 [15]. However, ABCC3 is the main ABCC protein overexpressed in PDAC [28], with other ABCC members being detected mainly at the mRNA level. Our own prior work reconfirmed these observations and demonstrated that PDAC cell lines exhibit the strongest expression of ABCC3, whereas low expression of ABCC1 was detected [5]. Therefore, the possible effects of ABCC1 inhibition by MCI-715 would be expected to be marginal. In addition, we show that MCI-715 has an increased efficacy in cells with high expression of ABCC3 (CAFs) compared to low-ABCC3 expressing (BJ). Similarly, low-ABCC3 expressing PDAC cell line, SW1990, showed minimal responsiveness to MCI-715. These findings further support the action of MCI-715 towards ABCC3 in these cells. However, we cannot exclude the potential interactions of MCI-715 with other targets and more studies are necessary to fully evaluate the action of this small molecule, although the chemical relatedness to sulindac and its lack of COX inhibition support a mechanism involving ABCC inhibition. Our results show that ABCC3 likely plays a role in tumour-stroma interactions and that ABCC3 inhibition with MCI-715 is a valid strategy for restraining the cancer-favouring role of the tumour microenvironment. Previous attempts in targeting PDAC stroma, although promising in vitro, failed in the clinical trials due to increased rates of metastatic spread [29, 30]. Our initial necropsy observations demonstrate not only lack of increased metastatic spread in the MCI-715 treated mice, but potentially reduced metastatic rats in these mice. However, further analysis is necessary to fully address this phenomenon. Nevertheless, the effectiveness and safety profile shown for MCI-715 in the KPC mouse model of pancreatic cancer support further evaluation of MCI-715 as therapeutic agent in PDAC treatment.

## Conclusions

In conclusion, we provide evidence of the pharmacological potential of ABCC3 inhibition with a small molecule inhibitor, MCI-715, to block PDAC progression in vivo without discernible toxicity. The clinical relevance of our results is emphasized by the fact that efficacy was demonstrated in both patient derived xenografts as well as KPC mice, a transgenic mouse model of PDAC that is the most predictive for pre-clinical evaluation of anti-tumorigenic agents for PDAC. In addition, sulindac, the scaffold on which MCI-715 was developed, is already approved for medical use, suggesting the possibility of rapid development down the translational path. Moreover, the oral bioavailability of MCI-715 presents an opportunity for a non-invasive and tolerable therapeutic approach. Overall, our findings support further preclinical validation and development of MCI-715 and related analogues for the treatment of pancreatic cancer.

## Additional file

Additional file 1: **Figure S1.** MCI-715- a specific inhibitor of ABCC3. **Figure S2.** Pharmacological inhibition of ABCC3 with MCI-715 reduces the activity of pSTAT3 Y705 and HIF1α. **Figure S3.** MCI-715 treatment in the animal models of PDAC. **Figure S4.** MCI-715 treatment induces apoptosis in the KPC primary cell line. **Figure S5.** Modulation of ABCC3 activity influences the viability of epithelial and stromal PDAC cells. (ZIP 4873 kb)

## Data Availability

The data that support the findings of this study are available from the corresponding author upon reasonable request.

## Abbreviations

CAFs: Cancer-associated fibroblasts
CMC: Carboxymethyl cellulose
COX: Cyclooxygenase
EGFR: Epidermal Growth Factor Receptor
HIF1α: Hypoxia Inducible Factor 1 Subunit Alpha
LPI: lysophosphatidylinositol
PDAC: Pancreatic Ductal Adenocarcinoma
STAT3: Signal Transducer and Activator of Transcription 3
αSMA: α Smooth Actin
