# Supplementary figures and images for: Pharmacological inhibition of ABCC3 slows tumour progression in animal models of pancreatic cancer
Source: J Exp Clin Cancer Res. 2019 Aug 5;38:312. doi: 10.1186/s13046-019-1308-7 (PMC6681491; doi:10.1186/s13046-019-1308-7)

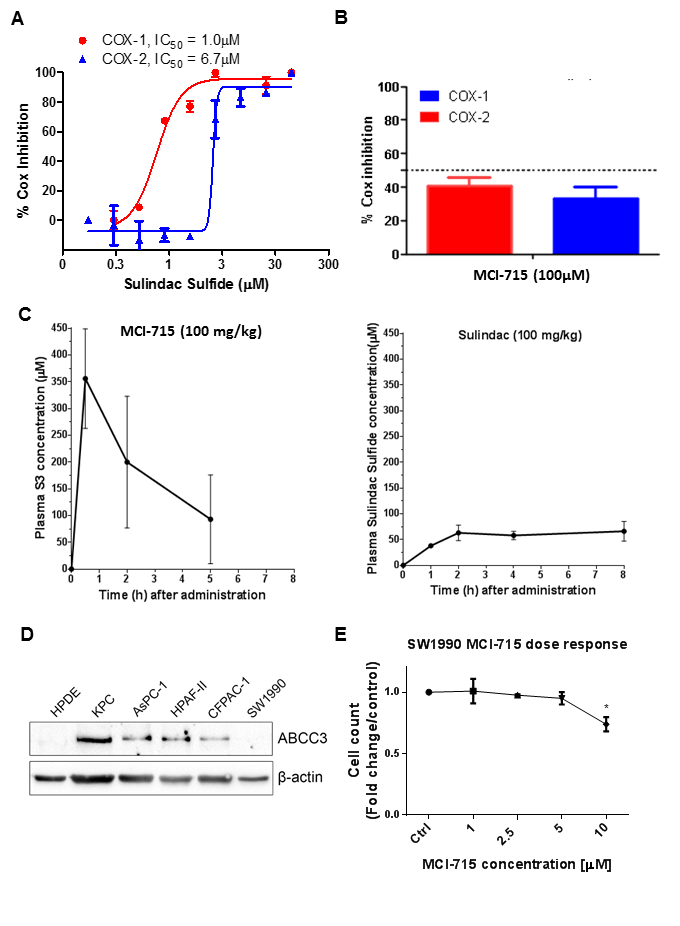

Supplement: Supplementary file 1 — Figure S1. MCI-715- a specific inhibitor of ABCC3. Figure S2. Pharmacological inhibition of ABCC3 with MCI-715 reduces the activity of pSTAT3 Y705 and HIF1α. Figure S3. MCI-715 treatment in the animal models of PDAC. Figure S4. MCI-715 treatment induces apoptosis in the KPC primary cell line. Figure S5. Modulation of ABCC3 activity influences the viability of epithelial and stromal PDAC cells. (ZIP 4873 kb) [file 13046_2019_1308_MOESM1_ESM.zip › Supplementart Figure 1.tif]

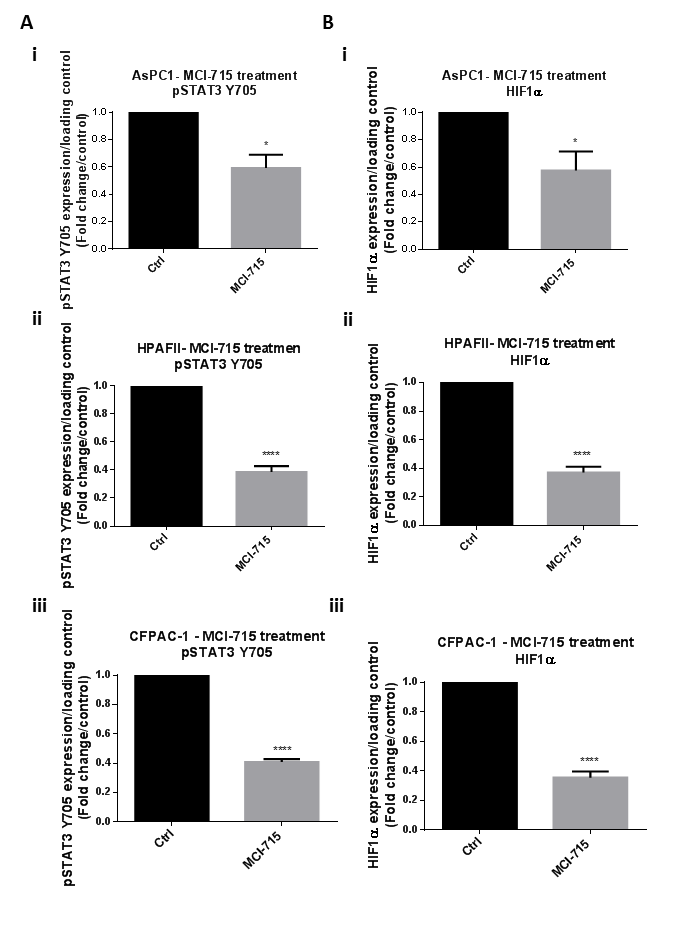

Supplement: Supplementary file 1 — Figure S1. MCI-715- a specific inhibitor of ABCC3. Figure S2. Pharmacological inhibition of ABCC3 with MCI-715 reduces the activity of pSTAT3 Y705 and HIF1α. Figure S3. MCI-715 treatment in the animal models of PDAC. Figure S4. MCI-715 treatment induces apoptosis in the KPC primary cell line. Figure S5. Modulation of ABCC3 activity influences the viability of epithelial and stromal PDAC cells. (ZIP 4873 kb) [file 13046_2019_1308_MOESM1_ESM.zip › Supplementary Figure 2.tif]

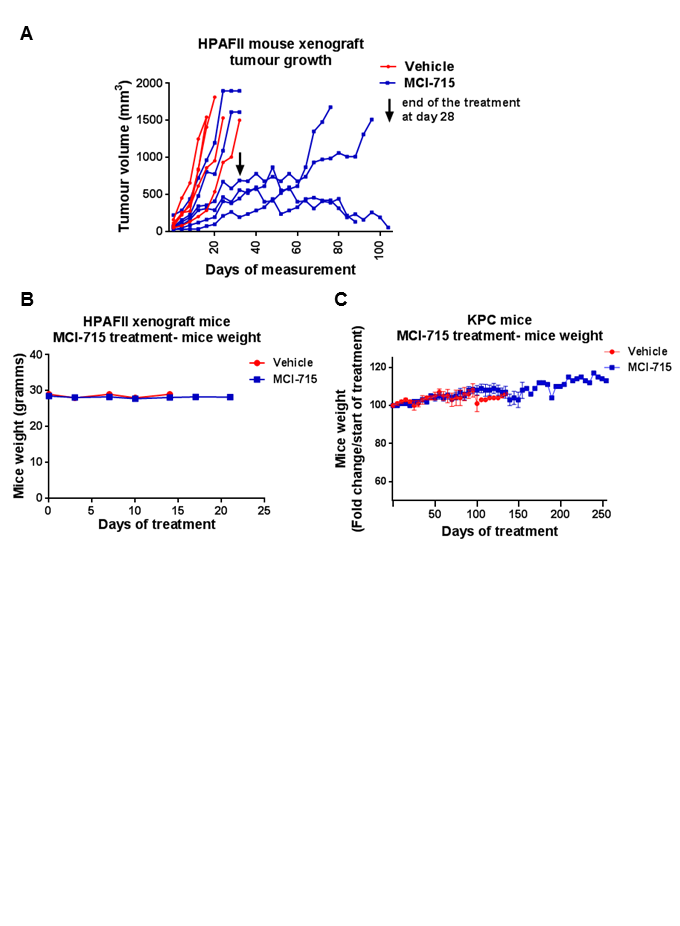

Supplement: Supplementary file 1 — Figure S1. MCI-715- a specific inhibitor of ABCC3. Figure S2. Pharmacological inhibition of ABCC3 with MCI-715 reduces the activity of pSTAT3 Y705 and HIF1α. Figure S3. MCI-715 treatment in the animal models of PDAC. Figure S4. MCI-715 treatment induces apoptosis in the KPC primary cell line. Figure S5. Modulation of ABCC3 activity influences the viability of epithelial and stromal PDAC cells. (ZIP 4873 kb) [file 13046_2019_1308_MOESM1_ESM.zip › Supplementary Figure 3.tif]
